# *Saccharomyces cerevisiae* Response to Magnetic Stress: Role of a Protein Corona in Stable Biosynthesis of Silver Nanoparticles

**DOI:** 10.3390/microorganisms14010178

**Published:** 2026-01-14

**Authors:** Atika Ahmad, Jahirul Ahmed Mazumder, Wafa AbuShar, Emilia Ouies, Ashif Yasin Sheikh, David Sheehan

**Affiliations:** Department of Chemistry, Khalifa University of Science and Technology, Abu Dhabi 127788, United Arab Emirates; 100059886@ku.ac.ae (A.A.); jahirul.mazumder@ku.ac.ae (J.A.M.); 100060603@ku.ac.ae (W.A.); emilia.oueis@ku.ac.ae (E.O.); ashif.shaikh@ku.ac.ae (A.Y.S.)

**Keywords:** *Saccharomyces cerevisiae*, green synthesis, silver nanoparticles, static magnetic field, transmission electron microscope

## Abstract

*Saccharomyces cerevisiae* was cultured under the influence of static magnetic fields (SMFs) to assess their impact on the biosynthesis of silver nanoparticles (AgNPs). Cell-free media derived from SMF-exposed cultures facilitated the formation of AgNPs, with a significant reduction in nanoparticle size observed at an optimal field strength of 7 mT. AgNPs synthesized under SMF conditions exhibited smaller crystalline structures than those produced in control media, as evidenced by dynamic light scattering (DLS) and transmission electron microscopy (TEM) measurements. Over a 75-day period, SMF-exposed AgNPs demonstrated enhanced stability, as determined by DLS and polydispersity index (PDI) assessments. Further analysis through sodium dodecyl-sulfate polyacrylamide gel electrophoresis (SDS-PAGE) and Fourier transform infrared spectroscopy (FTIR) suggested the formation of a protein corona on the AgNPs in SMF-treated samples, which likely inhibits agglomeration and enhances long-term stability. These findings indicate that SMF-induced stress in *S. cerevisiae* triggers the secretion of specific proteins that contribute to the stabilization of AgNPs, providing a novel approach to controlling nanoparticle synthesis and stability through magnetic field exposure.

## 1. Introduction

The budding yeast *Saccharomyces cerevisiae* possesses versatile biomolecular responses to external stressors [1,2,3]. Due to these robust systems, *S. cerevisiae* has emerged as an essential model organism for elucidating eukaryotic stress mechanisms [4,5]. Common stress factors such as decreased osmotic pressure [6,7], oxidative stress [8], extreme temperatures [9,10], and limited nutrient availability [11] trigger cellular responses in yeast cells. These responses often involve reorganizing metabolic pathways to mitigate the threat of external stressors. For example, *S. cerevisiae* responds to osmotic stress by switching its metabolism to accumulate glycerol [12,13]. The expression of heat shock proteins (Hsps) is significantly upregulated in several forms of stress [14,15]. Hsp12p, specifically, plays an important role in protecting the cellular organization of S. cerevisiae by acting as a plasticizer in the cell wall [16,17,18,19].

Metal nanoparticles have received significant attention due to their unique optical, surface, and antimicrobial characteristics [20,21,22,23,24,25]. Due to their extremely small size (diameters < 100 nm), they can sometimes agglomerate and lose their stability, resulting in a loss of functionality. It is desirable to obtain monodispersed, uniform, crystalline, and stable nanoparticles [26]. Synthesis of metal nanoparticles can be achieved through chemical and physical methods, which usually necessitate significant energy inputs and often have undesirable environmental consequences [27]. In contrast, green chemistry approaches utilize microorganisms and plants to reduce the accumulation and production of toxic chemicals in the environment [28,29]. Silver nanoparticles (AgNPs) are the most commercially valuable class of metallic nanoparticles due to their broad spectrum of antimicrobial properties [24,30,31,32,33,34]. Their conventional synthesis route requires toxic solvents and by-products, posing occupational health and environmental risks [35]. Achieving silver nanoparticles with defined size distribution, dispersity, crystallinity, and stability is technically challenging [36,37,38,39,40]. Green synthesis offers increased stability, efficient scale-up, biocompatibility, low energy requirements, and no toxic waste. Bacteria, fungi, and plants have inherent reduction mechanisms that can reduce metal salts to nanoparticles and stabilize them via coating with biomolecules such as proteins [41,42,43].

Static magnetic fields (SMFs) increasingly permeate the terrestrial environment due to the widespread contemporary use of personal electronic devices. Systematic investigations into the effects of SMF exposure and stress induction in model organisms such as yeast may provide deeper insights into their associated biological interactions and long-term effects. Relatively few studies have investigated the impact of magnetic stress on *S. cerevisiae*. Previous research has reported decreased viability and growth rate after exposing yeast cells to low-intensity magnetic fields [44], excessive growth suppression at field intensities of up to 250 mT [45], and oxidative stress leading to cell damage [46]. Magnetic stress has also been reported to increase transcript levels of specific genes associated with stress response [47]. Recently, it was observed that SMFs resulted in the induction of oxidative stress and the production of reactive oxygen species (ROS) [48]. Such studies offer potential insights into the effects of magnetic stress on *S. cerevisiae* [49,50]. Yeast offers simpler encapsulation mechanisms and secretion of biomolecules, which can stabilize synthesized nanoparticles [51,52,53]. Protein-rich layers coating nanoparticles are referred to as the protein “corona” (PC) [54,55,56]. The action of external stressors on yeast, such as heat shock, oxidative stress, and magnetic stress, can influence the corona’s composition. Effects induced by magnetic stress include the overexpression of stress genes and the altered expression of early response genes. Recent studies, such as those by Kthiri et al. (2019), observed that SMFs resulted in oxidative stress and ROS production [57]. These findings offer insights into the effects of magnetic stress on *S. cerevisiae* [58,59]. Expression of stress genes in the presence of SMFs demonstrates that cells perceive magnetic fields as a type of environmental stress [60,61,62]. The corona’s composition can be influenced by external stressors such as heat shock and oxidative and magnetic stress [63,64,65,66,67,68]. In this study, we explore the effects of low-intensity magnetic stress on *S. cerevisiae* in the biosynthesis of stable silver nanoparticles. We attribute SMF-associated stability to a stress-induced PC coating of the nanoparticles.

## 2. Materials and Methods

### 2.1. Chemical and Reagents

Silver nitrate (AgNO_3_), Tris(hydroxymethyl)aminomethane, and sodium dodecyl sulfate (SDS) were purchased from Sigma Aldrich, Life Science (Burlington, MA, USA). Precision Plus Protein™ Standard Plugs was purchased from Bio-Rad (Hercules, CA, USA). *Saccharomyces cerevisiae* (strain BY4741. Genotype: *MATa his 3Δ1 leu2Δ0 met15Δ0 ura3Δ0*) was obtained from ATCC. All other salts and chemicals used were of analytical grade and purchased from Sigma Aldrich, Life Science.

### 2.2. Synthesis of AgNP in the Presence of Magnetic Field

The *Saccharomyces cerevisiae* (strain BY4741. Genotype: *MATa his 3Δ1 leu2Δ0 met15Δ0 ura3Δ0*) obtained from ATCC were cultured in YPD broth (1% bacto-yeast extract, 2% bacto-peptone, 2% glucose). The cultures with increased growth were treated to a parallel and homogenous magnetic induction in the range of 0–10 mT, and a magnetic field was generated by Helmholtz coils (provided by Department of Physics, Khalifa University, Abu Dhabi, United Arab Emirates, UAE). The terrestrial magnetic field at the experimental location was 0.065 mT. The control cultures were identically prepared without exposure to a magnetic field. Cell-free supernatants were obtained from each culture by centrifugation (10,000× *g*), followed by filtration through Whatman Grade 2 filter paper (8 µm) (Whatman, UK). Subsequently, 1 mM of AgNO_3_ was added to the filtered supernatant. The supernatants were kept in the dark at room temperature for 24 h. AgNPs were collected through wash cycles and sequential centrifugation (10,000× *g*).

### 2.3. Characterization of Nanoparticles

#### 2.3.1. UV–Visible Absorbance Spectral Analysis

The biosynthesized AgNPs were detected through visual color change observation of the cell filtrate after treatment with 1 mM silver nitrate. The characterization of biosynthesized AgNPs was carried out through scanning in the range of 300–700 nm in a UV–visible spectrophotometer [Bruker UV-5500PC (Billerica, MA, USA)].

#### 2.3.2. Dynamic Light Scattering

A Zetasizer Nano S from Malvern Instruments Ltd. (Worcestershire, UK). was used to perform dynamic light scattering (DLS) analysis on 1 mg/mL AgNP samples in a low-volume quartz cuvette. The analysis was performed at 25 °C for 10 cycles. AgNPs synthesized at different field strengths (3–9 mT) were studied. AgNPs at 1 mg/mL concentration in Milli-Q water (Merck Millipore, Burlington, MA, USA) after sonication were used as a DLS study sample.

#### 2.3.3. Stability Assessment of AgNPs: Hydrodynamic Size and PDI Analysis

Both control and test samples of AgNPs were dissolved in ddH_2_O at a concentration of 1 mg/mL and subjected to DLS measurements over 75 days. The AgNPs were first suspended in a 6% SDS solution, then washed with 100 μL of Tris-HCl, centrifuged at 10,000× *g* for 10 min, and finally, the pellets were collected and resuspended in water. The resulting colloidal sample of AgNPs (Control and Test) was analyzed to assess changes in AgNP hydrodynamic size and polydispersity index (PDI).

#### 2.3.4. Fourier Transform Infrared Spectroscopy (FTIR)

AgNPs in the form of powder were used for analysis through Thermo Scientific Nicolet iS10 Fourier transform infrared (FTIR) spectrometry (Waltham, MA, USA). FTIR spectra of AgNPs (synthesized in the presence and absence of SMF) were recorded between 4000 to 500 cm^−1^ with a resolution of 4 cm^−1^. FTIR analysis was carried out to identify the possible functional groups present as capping agents on the surface of nanoparticles, which might be responsible for their enhanced stability.

#### 2.3.5. X-Ray Diffraction (XRD)

Powdered AgNP was used for XRD analysis in a Bruker D8 advance diffractometer (Bruker AXS, Karlsruhe, Germany) over a wide range of Bragg angles (20° ≤ 2θ ≤ 80°); the h, k, and l indices correlating to the 2-theta value were analyzed and matched with the corresponding Joint Committee on powder diffraction (JCPDS) file number.

#### 2.3.6. Transmission Electron Microscopy (TEM)

A transmission electron microscope (FEI Tecnai, Stanford, CA, USA) operating at 200 kV was used to study the size and shape of AgNPs. The sample preparation for TEM was carried out by drop-coating the diluted AgNPs on a carbon-coated copper grid.

### 2.4. Analysis of Protein Corona Around AgNP

#### Gel Electrophoresis

Tris–Glycine SDS-PAGE was performed according to Laemmli et al. [69]. The Bio-Rad Mini-PROTEAN system was used to hand-cast SDS-PAGE gels (Bio-Rad, Hercules, CA, USA). SDS-PAGE sample buffer (62.5 mmol L^−1^ Tris-HCl, pH 6.8; 2% *w*/*v* SDS) was added to 5 mg of AgNPs_(Control and Test)_, and the mixture was then boiled to denature and solubilize the proteins, followed by centrifugation at 1000× *g* for 15 min. For stacking and separating the proteins, 4% and 10% polyacrylamide gels were used, respectively. Until the dye front reached the lower end of the gel, electrophoresis was performed in an electrophoresis buffer at a steady current (60–100 V). Under agitation, Coomassie Blue R-250 (Thermo Fisher, Waltham, MA, USA) was used to stain the gels, followed by destaining (10% acetic acid, 50% methanol, and 40% H_2_O). Proteins were separated on a gradient mini-protean gel (Bio-Rad, 456–1094) at 60 mA and 100 V along with a Precision Plus Protein™ Standard Protein Plugs (Bio-Rad), unstained, (10–250 kD) molecular weight marker. Proteins were stained with Coomassie™ blue staining. All the experiments were run in triplicates to verify the reproducibility of the AgNP-protein content, general pattern, and band intensities on the gels.

### 2.5. Statistical Analysis

DLS experiments were carried out in three independent determinations, and all data are presented as mean ± standard deviation. The significance of the difference between the test and control was determined by t-test analysis in SPSS software version 25. Values of *p* < 0.05 were considered statistically significant.

## 3. Results

**Figure 1:**
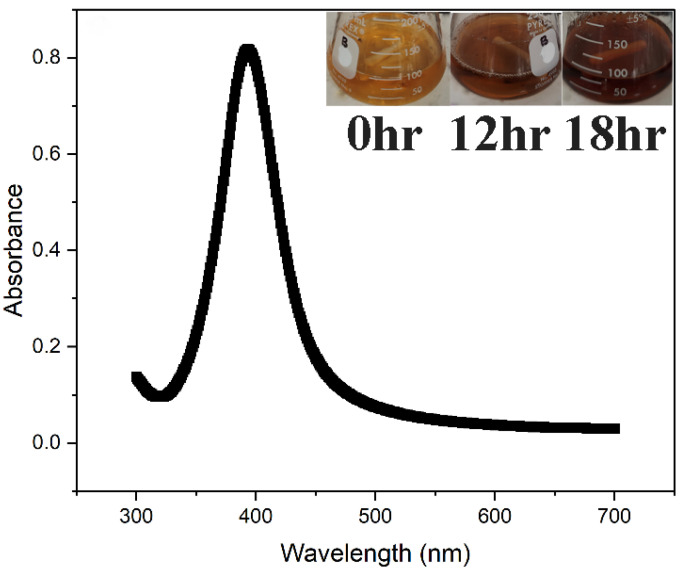
UV–visible spectrum of AgNP synthesized using *S. cerevisiae* under the influence of SMF. A maximum absorption peak was observed at 412 nm at 12 h of synthesis. The inset represents color change at different time intervals during the AgNP synthesis. The UV spectra of Blank (1 mM silver nitrate aqueous solution combined with YPD broth), Control (AgNP synthesis without SMF), and spectra of AgNP at 18 h are provided in Supplementary Files (Figures S1–S3).

Cell-free *S. cerevisiae* culture supernatants were employed in the current study to synthesize AgNPs. After 18 h of incubation at 25 °C, the color of the *S. cerevisiae* control supernatant changed from light yellow to brown with the addition of 1 mM silver nitrate aqueous solution (Figure 1). Reduction of Ag^+^ into AgNPs was followed by measuring sample UV–vis spectra. A surface plasmon resonance peak for AgNP was observed at 412 nm, consistent with the known absorbance peaks associated with AgNPs [70]. The inset of Figure 1 illustrates color changes from pale yellow to golden yellow and then to brown, signifying the biosynthesis of AgNP. Additionally, two controls were used; in one, the experimental set-up was maintained while the temperature was raised to 50 °C, and in the other, 1 mM silver nitrate aqueous solution was combined with YPD broth; no resonance peak was evident in either of these circumstances. Previously, it has been stated that the surface plasmon resonance-induced excitation of the solution’s color change from yellow to brown makes it possible to see the reduction of Ag^+^ into AgNPs quite clearly [71].

**Figure 2:**
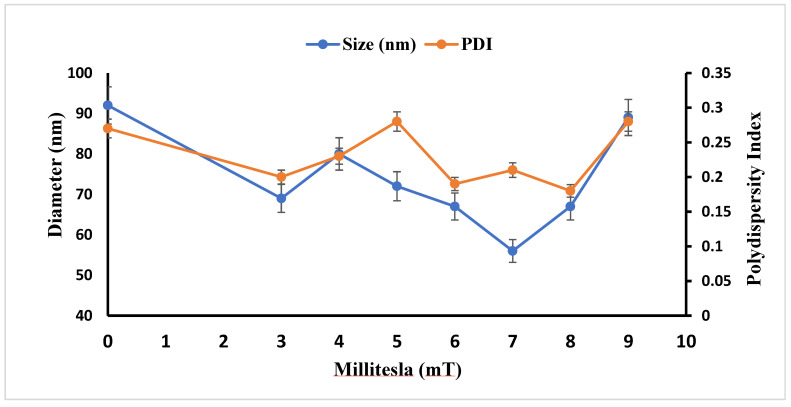
Dynamic light scattering graph of AgNP (synthesized using magnetic field strengths of 3–9 mT); here, 0 mT represents Control (no external magnetic field). The graph shows the minimal hydrodynamic size, and PDI was observed at 7 mT. The results are represented as three replicates (*n* = 3) ± SD. The significance level was maintained as a *p*-value < 0.05.

During the culture of *S. cerevisiae*, a range of magnetic field strengths, 3–9 mT, was employed. This was followed by AgNP biosynthesis to identify the optimal field strength yielding the most favorable hydrodynamic size and PDI. This analysis was performed using DLS. Figure 2 revealed the hydrodynamic size and PDI at various magnetic field strengths. DLS suggested that 7 mT was optimum for uniform size distribution and the most stable PDI.

**Figure 3:**
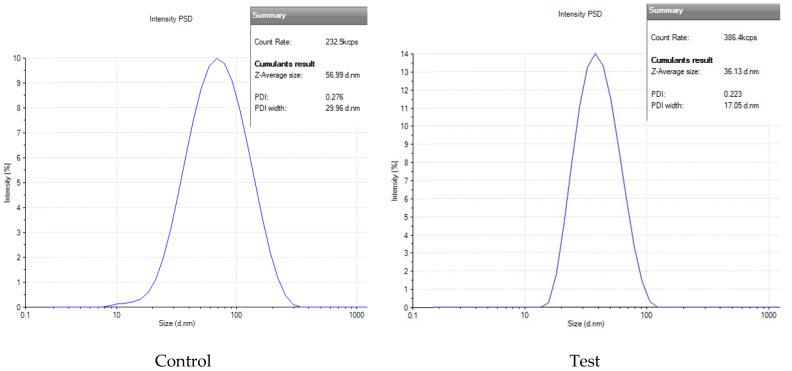
Dynamic light scattering graph showing Z-average size of freshly prepared AgNPs. The Z-average size of biosynthesized AgNPs in the presence of SMF (7 mT) is 36 ± 2 nm, while the Control (no SMF) shows a size of 57 ± 3 nm. Data represent the mean ± standard deviation (SD) of n = 3 biological replicates, where each biological replicate corresponds to an independent batch of synthesis of the nanoparticle. There was no difference observed in the technical replicates.

AgNPs were synthesized in the presence of SMF (test, 7 mT) and compared to a control sample without the magnetic field by DLS to further evaluate the effect of magnetic field strength. Figure 3 reveals that the test AgNP sample exhibited a hydrodynamic size of 36 nm, indicating relatively smaller nanoparticles. Furthermore, the PDI value of 0.22 indicated a nearly monodisperse particle distribution with minimal size variations. In comparison, the control sample, synthesized in the absence of the magnetic field, exhibited a larger hydrodynamic size of 57 nm. The PDI value of 0.27 indicated a moderate level of polydispersity, suggesting a broader size distribution compared to the test AgNP sample.

**Figure 4:**
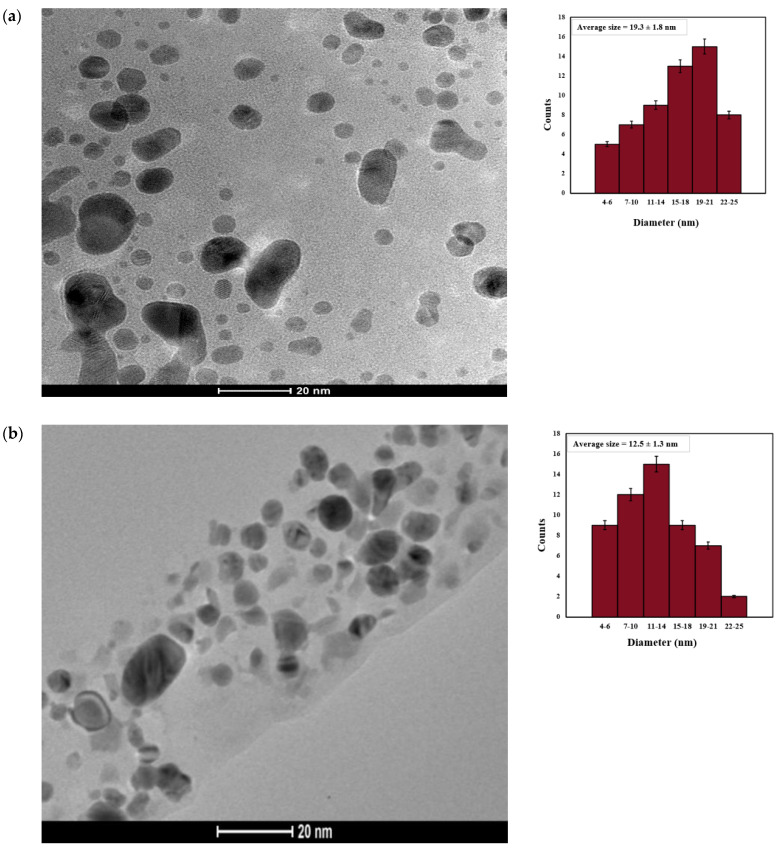
Transmission electron microscopy image of (**a**) AgNP_Control_ and (**b**) AgNP_Test_. Inset shows a histogram, showing mean average particles ranging between 19.3 ± 1.8 nm and 12.5 ± 1.3 nm for AgNP (Control and Test), respectively.

TEM was used to compare AgNP_control_ and AgNP_test_ (Figure 4). In the absence of SMF, the majority of AgNPs had diameters ranging from 10 to 25 nm (Figure 4a). However, in the presence of SMF, the majority of particles exhibited diameters in the range of 5 to 15 nm (Figure 4b). AgNP_Test_ nanoparticles were found to be more crystalline and smaller in size than AgNP_Control_ (Figure 4). Notably, nanoparticles appeared mostly spherical in both cases, but those synthesized under SMF conditions seemed to have a more uniform shape and size distribution.

**Figure 5:**
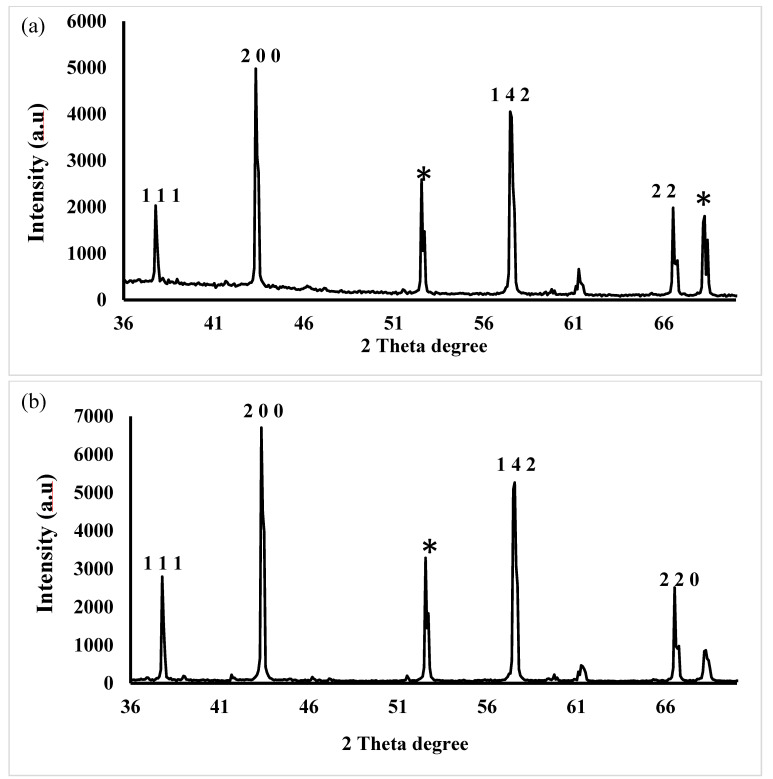
XRD patterns of (**a**) AgNP_Control_ and (**b**) AgNP_Test_. The 2-theta values were analyzed and matched with the corresponding JCPDS file number 04-0783. Peaks denoted by *, is possibly due to impurities present in the sample.

The crystalline nature and phase purity of both control and test samples of AgNPs were analyzed using XRD. Distinct peaks corresponding to crystallographic planes at (111), (200), (142), and (220) for both control (Figure 5a) and test samples (Figure 5b) were observed. These closely matched the standard JCPDS card number 04-0783. A few additional peaks in the control sample (denoted by *), possibly due to impurities, were not observed in test samples. The presence of well-defined peaks at specific crystallographic planes further confirms the successful biosynthesis of crystalline AgNPs.

**Figure 6:**
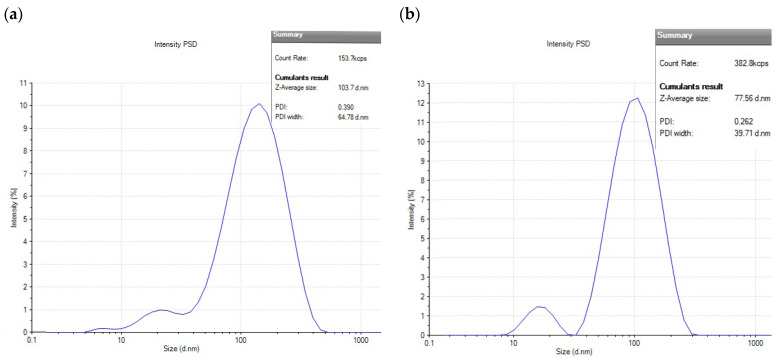
DLS analysis of (**a**) AgNP_Control_ and (**b**) AgNP_Test_ after washing with SDS and Tris buffer. SDS washing increased the size and PDI. Control: 57 ± 3 nm (PDI 0.27) to 103 ± 3 nm (PDI 0.39). Test: 36 ± 2 nm (PDI 0.22) to 77 ± 3 nm (PDI 0.26). Data are mean ± SD (n = 3).

A stability study of AgNPs revealed significant differences in hydrodynamic size and PDI between the SMF and control samples over 75 days. After the 75th day, it was observed that the size and PDI of the AgNP_Test_ sample remained relatively stable at approximately 63 nm. In contrast, the hydrodynamic size and PDI of the AgNP_Control_ sample displayed a consistent increase, eventually reaching 327 nm and a significantly higher PDI of 0.60, indicating pronounced polydispersity and agglomeration (Table 1). A protein removal procedure in which AgNPTest was washed with SDS was used to assess the role of protein adsorption on the hydrodynamic size and polydispersity index (PDI) of AgNPs. An analysis of Figure 6 demonstrated a significant increase in size and PDI for both the control and test samples after undergoing SDS washing. In the control sample, the histogram exhibited a broad peak centered at 103 nm with a PDI of 0.39 (increased from 56 nm with a PDI of 0.27), indicating agglomeration and reduced stability. Conversely, the test sample displayed a size of 77 nm and a PDI of 0.26 (increased from 36 nm with a PDI of 0.22).

**Figure 7:**
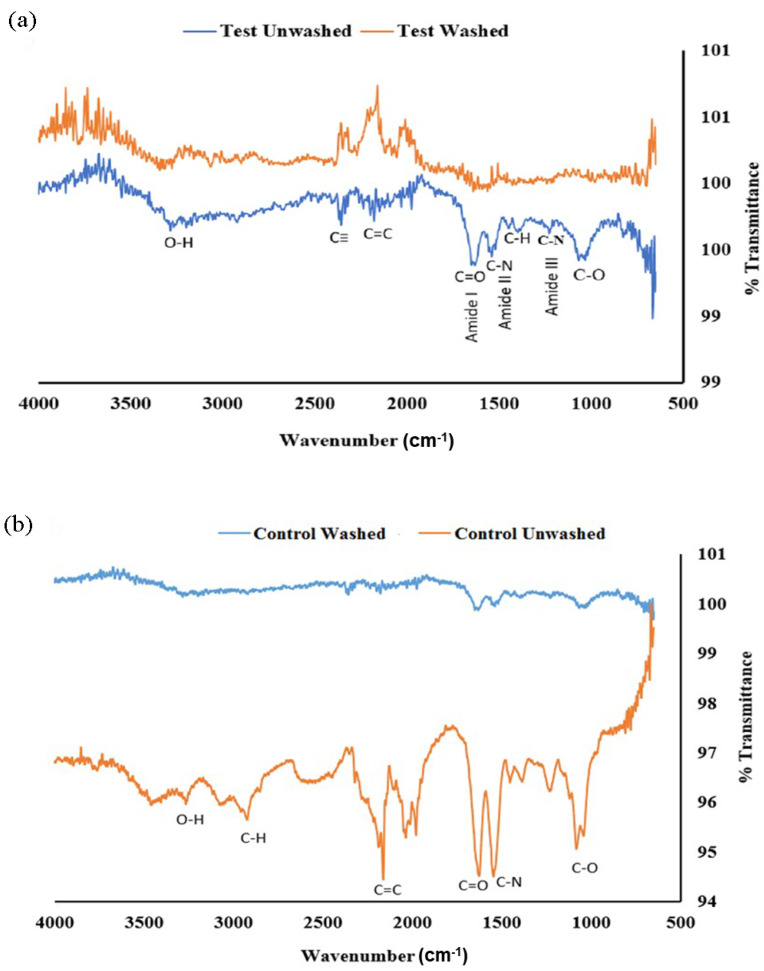
FTIR analysis of (**a**) AgNP_Test_ and (**b**) AgNP_Control_ revealed multiple peaks in the unwashed condition, indicating the presence of various functional groups on the nanoparticle surfaces.

FTIR analysis of AgNP_Test_, comparing SDS-washed and unwashed samples, revealed differences in the observed peaks. Figure 7a shows peaks corresponding to wave numbers 1635, 1550, and 1228 cm^−1^, representing Amide I, II, and III proteins, respectively. These were not present in the washed test sample. This suggests that the washing procedure successfully removed proteins contributing to these peaks. Additionally, other peaks, such as those associated with C=C, C≡C, and C-O bonds, were present in the unwashed condition, which was absent in the washed condition. Similar variations were observed in the AgNP_Control_ (Figure 7b), albeit with slight differences. These results indicate changes in the surface chemistry and composition of the AgNPs after washing with SDS.

**Figure 8:**
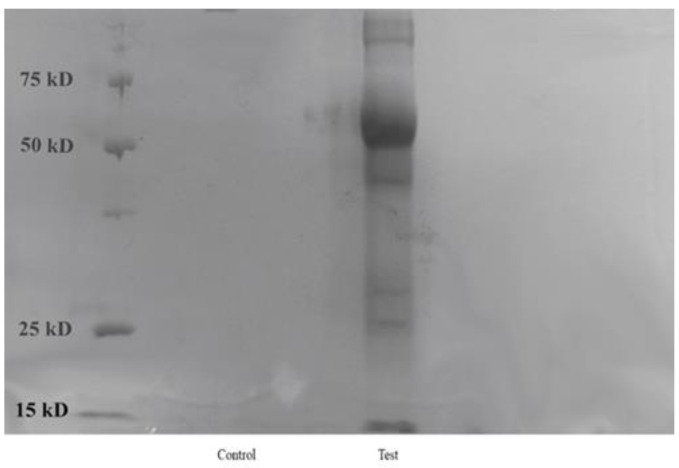
SDS-PAGE of proteins from AgNP_(control and Test)_ samples. The Test lane depicts multiple bands, which show the presence of multiple low molecular mass proteins on the surface of AgNP_(Test)_ samples.

To analyze adsorbed proteins on both the control and test samples of AgNPs, we boiled the nanoparticles in SDS-PAGE sample buffer to denature and solubilize proteins. We then centrifuged the mixture at 10,000× *g* for 15 min to pellet the nanoparticles, separating them from the supernatant. The supernatant, which contained the proteins, was loaded onto an SDS-PAGE gel for analysis. Low molecular (between 15 and 25 kD) and high molecular mass proteins (about 50 kD) can both be seen in the AgNP (Test) lane of the SDS-PAGE gel image, as illustrated in Figure 8.

## 4. Discussion

This study investigates the biosynthesis of stable AgNPs mediated by *Saccharomyces cerevisiae* under varying magnetic field strengths, aiming to understand how magnetic stress influences nanoparticle properties. *S. cerevisiae* is known to withstand a range of stressors, including magnetic fields, which can enhance protein expression and metabolic activity [72]. This study extends these findings by showing that magnetic fields influence the size and PDI of AgNPs during synthesis. Interaction between magnetic fields and biological systems may be attributed to alterations in cell membranes through ion channels and electron transport, which result in electro-activation and induction of several enzymes and metabolic pathways. Santos et al. (2010) reported that *S. cerevisiae* biomass and concentration of GSH increased significantly in cell cultures treated with magnetic fields at a strength of 25–34.3 mT. They also reported enhanced levels of GSH and peroxidase activity [73,74].

Our results indicate that a magnetic field strength of 7 mT optimized the properties of AgNPs, resulting in nanoparticles with desirable size and stability characteristics. The results demonstrate that varying magnetic field strengths significantly influence the hydrodynamic size and PDI of the AgNPs, indicating that the magnetic field acts as a stressor that induces specific stress proteins in *S. cerevisiae*. This, in turn, affects the stability of the nanoparticles by altering the protein corona. DLS and TEM results confirmed that the AgNPs synthesized under SMF had smaller and more uniform particle sizes compared to the control. Furthermore, the PC plays a role in maintaining nanoparticle stability [75,76,77]. This was demonstrated by the increased particle size and PDI following SDS washing, which removed the PC from the nanoparticles. The size and PDI increased more significantly in the control group than in the SMF-exposed group, underscoring the importance of the PC in stabilizing AgNPs. These findings align with previous studies, which have established the correlation between PC formation and nanoparticle stability, particularly in preventing agglomeration in biological environments [78,79,80,81,82]. SDS-PAGE and FTIR analyses further validated the presence of stress-induced proteins in the SMF-treated samples. Distinct protein profiles were observed between SMF-exposed and control AgNPs, with proteins associated with Amide I, II, and III bands present in the SMF-treated AgNPs. Differences in protein profiles were observed between SMF-exposed and control AgNPs, with proteins associated with Amide I, II, and III bands present in the SMF-treated AgNPs. The results underscore the importance of the PC in enhancing nanoparticle stability and size control under magnetic stress.

## 5. Conclusions

This study demonstrates that SMF acts as a stressor to *S. cerevisiae*, triggering a biochemical stress response. Specifically, SMF exposure induces the secretion of proteins that form a stabilizing PC around AgNPs, enhancing their stability over time. FTIR and SDS-PAGE analyses confirmed the presence of this corona in SMF-treated samples, which was absent in control groups. The results suggest that SMF-induced cellular stress in *S. cerevisiae* plays a crucial role in the biosynthesis and stabilization of AgNPs. However, further research is essential to delve deeper into the specific proteins forming the corona and to unravel the underlying mechanisms of SMF-induced changes. This future exploration could offer valuable insights into refining nanoparticle synthesis for various applications.

## Figures and Tables

**Table 1:** Represents the hydrodynamic size and PDI of AgNP_(Test)_ at 7 mT and AgNP_(Control)_ measured over 75 days using DLS. The results are represented as mean of three replicates (n = 3) ± SD. The significance level was maintained as a *p*-value < 0.05. Statistical data analysis was performed using an independent t-test to compare Test and Control. The *t*-test revealed a significant difference between the two groups, AgNP_(Control)_ and AgNP_(Test)_ (*p* = 0.0282), indicating a statistically significant effect.

Days	Size (nm) and PDI of AgNP_(Test)_	Size (nm) and PDI of AgNP_(Control)_
1	36 ± 1.5, 0.22	56 ± 3.2, 0.27
15	57 ± 1.76, 0.23	81 ± 3.8, 0.27
30	61 ± 1.54, 0.23	92 ± 4.1, 0.27
45	63 ± 2.1. 0.22	98 ± 3.9, 0.42
50	61 ± 2.3, 0.20	110 ± 2.7, 0.53
65	60.8 ± 1.87, 0.21	263 ± 5.3, 0.55
75	63.2 ± 1.01, 0.20	327 ± 4.21, 0.60

## Data Availability

The original contributions presented in the study are included in the article/Supplementary Material, further inquiries can be directed to the corresponding author.

## Supplementary Materials

The following supporting information can be downloaded at: https://www.mdpi.com/article/10.3390/microorganisms14010178/s1, Figure S1: UV-vis spectra of Blank, where 1 mM aqueous AgNO_3_ solution was mixed with YPD broth. No peak was observed; Figure S2: UV-vis spectra of Control, where AgNP synthesis was carried out using *S.cerevisiae* (without the influence of SMF). A broad peak was observed; Figure S3: UV-vis spectra at 18 h of AgNP synthesis. A broad peak was observed, indicating the formation of silver nanoparticles.

## References

[1] Mager W.H., Ferreira P.M. (1993). Stress Response of Yeast. Biochem. J..

[2] Jamieson D.J. (1998). Oxidative Stress Responses of the Yeast *Saccharomyces cerevisiae*. Yeast.

[3] Postaru M., Tucaliuc A., Cascaval D., Galaction A.-I. (2023). Cellular Stress Impact on Yeast Activity in Biotechnological Processes—A Short Overview. Microorganisms.

[4] Lopandic K. (2018). Saccharomyces Interspecies Hybrids as Model Organisms for Studying Yeast Adaptation to Stressful Environments. Yeast.

[5] Martorell P., Forment J.V., de Llanos R., Montón F., Llopis S., González N., Genovés S., Cienfuegos E., Monzó H., Ramón D. (2011). Use of *Saccharomyces cerevisiae* and *Caenorhabditis elegans* as Model Organisms to Study the Effect of Cocoa Polyphenols in the Resistance to Oxidative Stress. J. Agric. Food Chem..

[6] Mager W.H., Siderius M. (2002). Novel Insights into the Osmotic Stress Response of Yeast. FEMS Yeast Res..

[7] Varela J.C., Praekelt U.M., Meacock P.A., Planta R.J., Mager W.H. (1995). The *Saccharomyces cerevisiae* HSP12 Gene Is Activated by the High-Osmolarity Glycerol Pathway and Negatively Regulated by Protein Kinase A. Mol. Cell. Biol..

[8] Jamieson D.J. (1995). The Effect of Oxidative Stress on *Saccharomyces cerevisiae*. Redox Rep..

[9] Ruis H., Schüller C. (1995). Stress Signaling in Yeast. Bioessays.

[10] Jenkins G.M., Richards A., Wahl T., Mao C., Obeid L., Hannun Y. (1997). Involvement of Yeast Sphingolipids in the Heat Stress Response of *Saccharomyces cerevisiae* *. J. Biol. Chem..

[11] Swinnen E., Wanke V., Roosen J., Smets B., Dubouloz F., Pedruzzi I., Cameroni E., De Virgilio C., Winderickx J. (2006). Rim15 and the Crossroads of Nutrient Signalling Pathways in *Saccharomyces cerevisiae*. Cell Div..

[12] Saito H., Posas F. (2012). Response to Hyperosmotic Stress. Genetics.

[13] Cavelius P., Engelhart-Straub S., Biewald A., Haack M., Awad D., Brueck T., Mehlmer N. (2023). Adaptation of Proteome and Metabolism in Different Haplotypes of *Rhodosporidium toruloides* during Cu(I) and Cu(II) Stress. Microorganisms.

[14] Craig E.A., Weissman J.S., Horwich A.L. (1994). Heat Shock Proteins and Molecular Chaperones: Mediators of Protein Conformation and Turnover in the Cell. Cell.

[15] Piper P.W. (1995). The Heat Shock and Ethanol Stress Responses of Yeast Exhibit Extensive Similarity and Functional Overlap. FEMS Microbiol. Lett..

[16] Karreman R.J., Lindsey G.G. (2005). A Rapid Method to Determine the Stress Status of *Saccharomyces cerevisiae* by Monitoring the Expression of a Hsp12: Green Fluorescent Protein (GFP) Construct under the Control of the Hsp12 Promoter. SLAS Discov..

[17] Sales K., Brandt W., Rumbak E., Lindsey G. (2000). The LEA-like Protein HSP 12 in *Saccharomyces cerevisiae* Has a Plasma Membrane Location and Protects Membranes against Desiccation and Ethanol-Induced Stress. Biochim. Et Biophys. Acta BBA Biomembr..

[18] Motshwene P., Karreman R., Kgari G., Brandt W., Lindsey G. (2004). LEA (Late Embryonic Abundant)-like Protein Hsp 12 (Heat-Shock Protein 12) Is Present in the Cell Wall and Enhances the Barotolerance of the Yeast *Saccharomyces cerevisiae*. Biochem. J..

[19] Karreman R.J., Dague E., Gaboriaud F., Quilès F., Duval J.F., Lindsey G.G. (2007). The Stress Response Protein Hsp12p Increases the Flexibility of the Yeast *Saccharomyces cerevisiae* Cell Wall. Biochim. Et Biophys. Acta BBA Proteins Proteom..

[20] Hirata Y., Andoh T., Asahara T., Kikuchi A. (2003). Yeast Glycogen Synthase Kinase-3 Activates Msn2p-Dependent Transcription of Stress Responsive Genes. Mol. Biol. Cell.

[21] Boy-Marcotte E., Lagniel G., Perrot M., Bussereau F., Boudsocq A., Jacquet M., Labarre J. (1999). The Heat Shock Response in Yeast: Differential Regulations and Contributions of the Msn2p/Msn4p and Hsf1p Regulons. Mol. Microbiol..

[22] Martinez-Pastor M.T., Marchler G., Schüller C., Marchler-Bauer A., Ruis H., Estruch F. (1996). The *Saccharomyces cerevisiae* Zinc Finger Proteins Msn2p and Msn4p Are Required for Transcriptional Induction through the Stress Response Element (STRE). EMBO J..

[23] Smith A., Ward M.P., Garrett S. (1998). Yeast PKA Represses Msn2p/Msn4p-dependent Gene Expression to Regulate Growth, Stress Response and Glycogen Accumulation. EMBO J..

[24] Perwez M., Mazumder J.A., Noori R., Sardar M. (2021). Magnetic combi CLEA for inhibition of bacterial biofilm: A green approach. Int. J. Biol. Macromol..

[25] Estruch F. (2000). Stress-Controlled Transcription Factors, Stress-Induced Genes and Stress Tolerance in Budding Yeast. FEMS Microbiol. Rev..

[26] Morano K.A., Grant C.M., Moye-Rowley W.S. (2012). The Response to Heat Shock and Oxidative Stress in *Saccharomyces cerevisiae*. Genetics.

[27] Slavin Y.N., Asnis J., Häfeli U.O., Bach H. (2017). Metal Nanoparticles: Understanding the Mechanisms behind Antibacterial Activity. J. Nanobiotechnol..

[28] Jamkhande P.G., Ghule N.W., Bamer A.H., Kalaskar M.G. (2019). Metal Nanoparticles Synthesis: An Overview on Methods of Preparation, Advantages and Disadvantages, and Applications. J. Drug Deliv. Sci. Technol..

[29] Lok C.-N., Ho C.-M., Chen R., He Q.-Y., Yu W.-Y., Sun H., Tam P.K.-H., Chiu J.-F., Che C.-M. (2007). Silver Nanoparticles: Partial Oxidation and Antibacterial Activities. JBIC J. Biol. Inorg. Chem..

[30] Ahmad A., Qurashi A., Sheehan D. (2023). Nano Packaging—Progress and Future Perspectives for Food Safety, and Sustainability. Food Packag. Shelf Life.

[31] Niu B., Zhang G. (2023). Effects of Different Nanoparticles on Microbes. Microorganisms.

[32] Sánchez-Rojas T., Espinoza-Culupú A., Ramírez P., Iwai L.K., Montoni F., Macedo-Prada D., Sulca-López M., Durán Y., Farfán-López M., Herencia J. (2022). Proteomic Study of Response to Copper, Cadmium, and Chrome Ion Stress in Yarrowia Lipolytica Strains Isolated from Andean Mine Tailings in Peru. Microorganisms.

[33] Fritz M., Chen X., Yang G., Lv Y., Liu M., Wehner S., Fischer C.B. (2024). Gold Nanoparticles Bioproduced in Cyanobacteria in the Initial Phase Opened an Avenue for the Discovery of Corresponding Cerium Nanoparticles. Microorganisms.

[34] Ali A., Shah T., Ullah R., Zhou P., Guo M., Ovais M., Tan Z., Rui Y. (2021). Review on Recent Progress in Magnetic Nanoparticles: Synthesis, Characterization, and Diverse Applications. Front. Chem..

[35] Alabdallah N.M., Kotb E. (2023). Antimicrobial Activity of Green Synthesized Silver Nanoparticles Using Waste Leaves of *Hyphaene thebaica* (Doum Palm). Microorganisms.

[36] Sudagar A.J., Rangam N.V., Ruszczak A., Borowicz P., Tóth J., Kövér L., Michałowska D., Roszko M.Ł., Noworyta K.R., Lesiak B. (2021). Valorization of Brewery Wastes for the Synthesis of Silver Nanocomposites Containing Orthophosphate. Nanomaterials.

[37] Bertelà F., Marsotto M., Meneghini C., Burratti L., Maraloiu V.-A., Iucci G., Venditti I., Prosposito P., D’Ezio V., Persichini T. (2021). Biocompatible Silver Nanoparticles: Study of the Chemical and Molecular Structure, and the Ability to Interact with Cadmium and Arsenic in Water and Biological Properties. Nanomaterials.

[38] Jalal M., Ansari M.A., Alzohairy M.A., Ali S.G., Khan H.M., Almatroudi A., Raees K. (2018). Biosynthesis of Silver Nanoparticles from Oropharyngeal *Candida glabrata* Isolates and Their Antimicrobial Activity against Clinical Strains of Bacteria and Fungi. Nanomaterials.

[39] Jahan I., Matpan Bekler F., Tunç A., Güven K. (2024). The Effects of Silver Nanoparticles (AgNPs) on Thermophilic Bacteria: Antibacterial, Morphological, Physiological and Biochemical Investigations. Microorganisms.

[40] Alghofaily M., Alfraih J., Alsaud A., Almazrua N., Sumague T.S., Auda S.H., Alsalleeh F. (2024). The Effectiveness of Silver Nanoparticles Mixed with Calcium Hydroxide against Candida Albicans: An Ex Vivo Analysis. Microorganisms.

[41] Elzahaby D.A., Farrag H.A., Haikal R.R., Alkordi M.H., Abdeltawab N.F., Ramadan M.A. (2023). Inhibition of Adherence and Biofilm Formation of *Pseudomonas aeruginosa* by Immobilized ZnO Nanoparticles on Silicone Urinary Catheter Grafted by Gamma Irradiation. Microorganisms.

[42] Kthiri A., Hamimed S., Othmani A., Landoulsi A., O’Sullivan S., Sheehan D. (2021). Novel Static Magnetic Field Effects on Green Chemistry Biosynthesis of Silver Nanoparticles in *Saccharomyces cerevisiae*. Sci. Rep..

[43] Mazumder J.A., Ahmad R., Sardar M. (2016). Reusable Magnetic Nanobiocatalyst for Synthesis of Silver and Gold Nanoparticles. Int. J. Biol. Macromol..

[44] Ali M.A., Ahmed T., Wu W., Hossain A., Hafeez R., Islam Masum M.M., Wang Y., An Q., Sun G., Li B. (2020). Advancements in Plant and Microbe-Based Synthesis of Metallic Nanoparticles and Their Antimicrobial Activity against Plant Pathogens. Nanomaterials.

[45] Kthiri A., Hamimed S., Tahri W., Landoulsi A., O’Sullivan S., Sheehan D. (2024). Impact of Silver Ions and Silver Nanoparticles on Biochemical Parameters and Antioxidant Enzyme Modulations in *Saccharomyces cerevisiae* under Co-Exposure to Static Magnetic Field: A Comparative Investigation. Int. Microbiol..

[46] Shanmugam J., Dhayalan M., Savaas Umar M.R., Gopal M., Ali Khan M., Simal-Gandara J., Cid-Samamed A. (2022). Green Synthesis of Silver Nanoparticles Using *Allium cepa* var. Aggregatum Natural Extract: Antibacterial and Cytotoxic Properties. Nanomaterials.

[47] Yang Q., Guo J., Long X., Pan C., Liu G., Peng J. (2023). Green Synthesis of Silver Nanoparticles Using Jasminum Nudiflorum Flower Extract and Their Antifungal and Antioxidant Activity. Nanomaterials.

[48] Kshirsagar P.G., De Matteis V., Pal S., Sangaru S.S. (2023). Silver–Gold Alloy Nanoparticles (AgAu NPs): Photochemical Synthesis of Novel Biocompatible, Bimetallic Alloy Nanoparticles and Study of Their In Vitro Peroxidase Nanozyme Activity. Nanomaterials.

[49] Islam R., Sun L., Zhang L. (2021). Biomedical Applications of Chinese Herb-Synthesized Silver Nanoparticles by Phytonanotechnology. Nanomaterials.

[50] Iravani S. (2011). Green Synthesis of Metal Nanoparticles Using Plants. Green. Chem..

[51] Hussain I., Singh N.B., Singh A., Singh H., Singh S.C. (2016). Green Synthesis of Nanoparticles and Its Potential Application. Biotechnol. Lett..

[52] Tomah A.A., Zhang Z., Alamer I.S.A., Khattak A.A., Ahmed T., Hu M., Wang D., Xu L., Li B., Wang Y. (2023). The Potential of Trichoderma-Mediated Nanotechnology Application in Sustainable Development Scopes. Nanomaterials.

[53] Novák J., Strašák L., Fojt L., Slaninová I., Vetterl V. (2007). Effects of Low-Frequency Magnetic Fields on the Viability of Yeast *Saccharomyces cerevisiae*. Bioelectrochemistry.

[54] Iwasaka M., Ikehata M., Miyakoshi J., Ueno S. (2004). Strong Static Magnetic Field Effects on Yeast Proliferation and Distribution. Bioelectrochemistry.

[55] Friedl A.A., Kiechle M., Fellerhoff B., Eckardt-Schupp F. (1998). Radiation-Induced Chromosome Aberrations in *Saccharomyces cerevisiae*: Influence of DNA Repair Pathways. Genetics.

[56] Weisbrot D.R., Khorkova O., Lin H., Henderson A.S., Goodman R. (1993). The Effect of Low Frequency Electric and Magnetic Fields on Gene Expression in *Saccharomyces cerevisiae*. Bioelectrochem. Bioenerg..

[57] Kthiri A., Hidouri S., Wiem T., Jeridi R., Sheehan D., Landouls A. (2019). Biochemical and Biomolecular Effects Induced by a Static Magnetic Field in *Saccharomyces cerevisiae*: Evidence for Oxidative Stress. PLoS ONE.

[58] Robinson J.R., Isikhuemhen O.S., Anike F.N. (2021). Fungal–Metal Interactions: A Review of Toxicity and Homeostasis. J. Fungi.

[59] Robinson J.R., Isikhuemhen O.S., Anike F.N., Subedi K. (2022). Physiological Response of *Saccharomyces cerevisiae* to Silver Stress. J. Fungi.

[60] Boroumand Moghaddam A., Namvar F., Moniri M., Tahir P.M., Azizi S., Mohamad R. (2015). Nanoparticles Biosynthesized by Fungi and Yeast: A Review of Their Preparation, Properties, and Medical Applications. Molecules.

[61] Jha A.K., Prasad K., Kulkarni A.R. (2008). Yeast Mediated Synthesis of Silver Nanoparticles. Int. J. Nanosci. Nanotechnol..

[62] Yang Y., Yang G., Li Z.-J., Liu Y.-S., Gao X.-D., Nakanishi H. (2023). Studies on the Proteinaceous Structure Present on the Surface of the *Saccharomyces cerevisiae* Spore Wall. J. Fungi.

[63] Kopac T. (2021). Protein Corona, Understanding the Nanoparticle–Protein Interactions and Future Perspectives: A Critical Review. Int. J. Biol. Macromol..

[64] Miceli E., Kuropka B., Rosenauer C., Osorio Blanco E.R., Theune L.E., Kar M., Weise C., Morsbach S., Freund C., Calderón M. (2018). Understanding the Elusive Protein Corona of Thermoresponsive Nanogels. Nanomedicine.

[65] Carrillo-Carrion C., Carril M., Parak W.J. (2017). Techniques for the Experimental Investigation of the Protein Corona. Curr. Opin. Biotechnol..

[66] Peigneux A., Glitscher E.A., Charbaji R., Weise C., Wedepohl S., Calderón M., Jimenez-Lopez C., Hedtrich S. (2020). Protein Corona Formation and Its Influence on Biomimetic Magnetite Nanoparticles. J. Mater. Chem. B.

[67] Ghodbane S., Lahbib A., Sakly M., Abdelmelek H. (2013). Bioeffects of Static Magnetic Fields: Oxidative Stress, Genotoxic Effects, and Cancer Studies. BioMed Res. Int..

[68] Toledano M.B., Delaunay A., Biteau B., Spector D., Azevedo D., Hohmann S., Mager W.H. (2003). Oxidative Stress Responses in Yeast. Yeast Stress Responses.

[69] Laemmli U.K. (1970). Cleavage of Structural Proteins During the Assembly of the Head of Bacteriophage T4. Nature.

[70] Smitha S.L., Nissamudeen K.M., Philip D., Gopchandran K.G. (2008). Studies on Surface Plasmon Resonance and Photoluminescence of Silver Nanoparticles. Spectrochim. Acta Part. A Mol. Biomol. Spectrosc..

[71] Sun Y., Xia Y. (2003). Gold and Silver Nanoparticles: A Class of Chromophores with Colors Tunable in the Range from 400 to 750 Nm. Analyst.

[72] Gasch A.P., Spellman P.T., Kao C.M., Carmel-Harel O., Eisen M.B., Storz G., Botstein D., Brown P.O. (2000). Genomic Expression Programs in the Response of Yeast Cells to Environmental Changes. MBoC.

[73] Santos L.O., Alegre R.M., Garcia-Diego C., Cuellar J. (2010). Effects of Magnetic Fields on Biomass and Glutathione Production by the Yeast *Saccharomyces cerevisiae*. Process Biochem..

[74] Machado B.R., Silva P.G.P., Garda-Buffon J., Santos L.O. (2022). Magnetic Fields as Inducer of Glutathione and Peroxidase Production by *Saccharomyces cerevisiae*. Braz. J. Microbiol..

[75] Bashiri G., Padilla M.S., Swingle K.L., Shepherd S.J., Mitchell M.J., Wang K. (2023). Nanoparticle Protein Corona: From Structure and Function to Therapeutic Targeting. Lab Chip.

[76] Li X., He E., Jiang K., Peijnenburg W.J.G.M., Qiu H. (2021). The Crucial Role of a Protein Corona in Determining the Aggregation Kinetics and Colloidal Stability of Polystyrene Nanoplastics. Water Res..

[77] Hajipour M.J., Safavi-Sohi R., Sharifi S., Mahmoud N., Ashkarran A.A., Voke E., Serpooshan V., Ramezankhani M., Milani A.S., Landry M.P. (2023). An Overview of Nanoparticle Protein Corona Literature. Small.

[78] Lima T., Bernfur K., Vilanova M., Cedervall T. (2020). Understanding the Lipid and Protein Corona Formation on Different Sized Polymeric Nanoparticles. Sci. Rep..

[79] Meesaragandla B., Blessing D.O., Karanth S., Rong A., Geist N., Delcea M. (2023). Interaction of polystyrene nanoparticles with supported lipid bilayers: Impact of nanoparticle size and protein corona. Macromol. Biosci..

[80] Zhang H., Peng J., Li X., Liu S., Hu Z., Xu G., Wu R. (2018). A Nano-Bio Interfacial Protein Corona on Silica Nanoparticle. Colloids Surf. B Biointerfaces.

[81] Yu Q., Zhao L., Guo C., Yan B., Su G. (2020). Regulating Protein Corona Formation and Dynamic Protein Exchange by Controlling Nanoparticle Hydrophobicity. Front. Bioeng. Biotechnol..

[82] Xiao B., Liu Y., Chandrasiri I., Overby C., Benoit D.S.W. (2023). Impact of Nanoparticle Physicochemical Properties on Protein Corona and Macrophage Polarization. ACS Appl. Mater. Interfaces.

