# Supplementary material for: Saccharomyces cerevisiae Response to Magnetic Stress: Role of a Protein Corona in Stable Biosynthesis of Silver Nanoparticles
Source: Microorganisms. 2026 Jan 14;14(1):178. doi: 10.3390/microorganisms14010178 (PMC12844350; doi:10.3390/microorganisms14010178)
Supplement: Supplementary file 1 [file microorganisms-14-00178-s001.zip › microorganisms-3266984-supplementary.pdf]

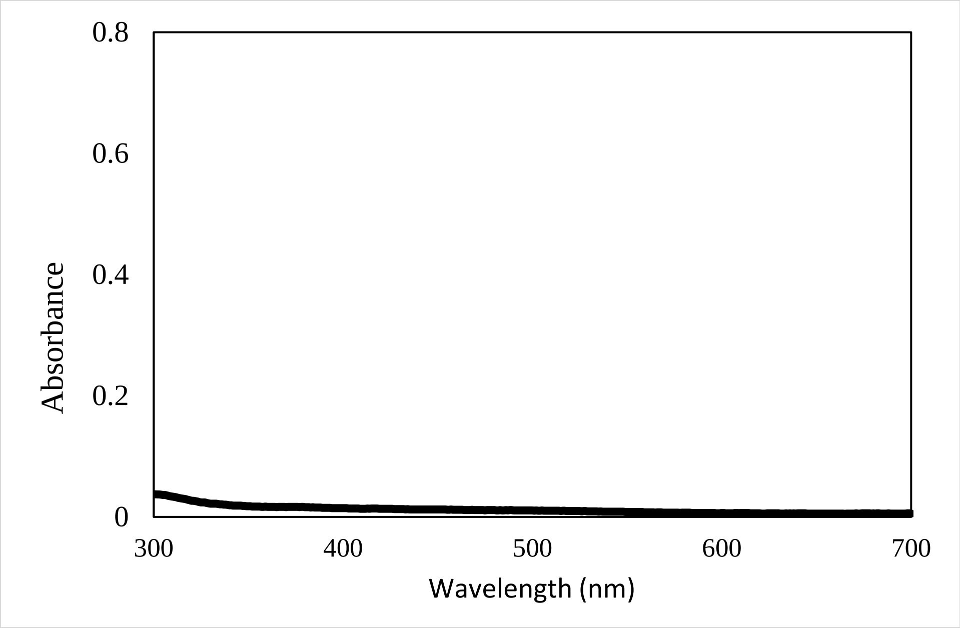

Figure S1: UV-Vis Spectra of Blank, where 1mM aqueous $AgNO_3$ solution was mixed with YPD broth. No peak was observed.

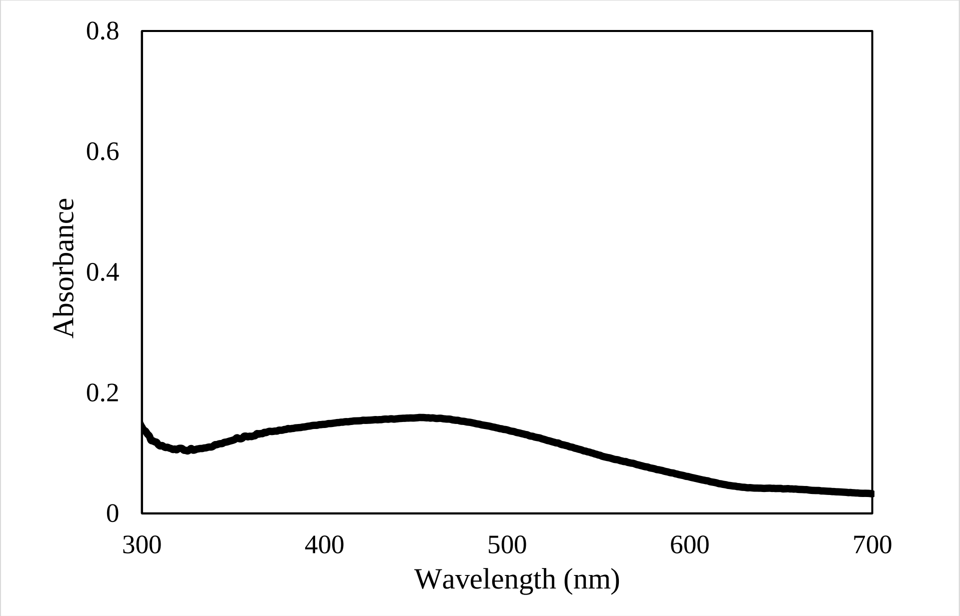

Figure S2: UV-Vis Spectra of Control, where AgNP synthesis was carried out using *S.cerevisiae* (Without the influence of SMF). A broad peak was observed.

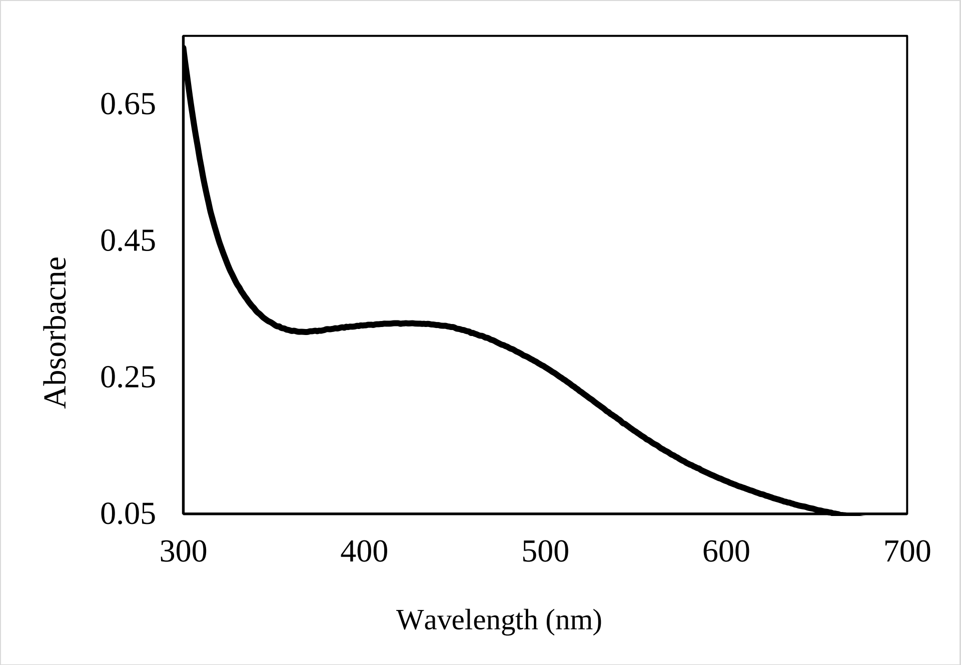

Figure S3: UV-Vis spectra at 18 hours of AgNP synthesis. A broad peak was observed, indicating the formation of Silver nanoparticles.
